# Simple Model for Alkali Leaching from Geopolymers: Effects of Raw Materials and Acetic Acid Concentration on Apparent Diffusion Coefficient

**DOI:** 10.3390/ma14061425

**Published:** 2021-03-15

**Authors:** Neven Ukrainczyk

**Affiliations:** Institute of Construction and Building Materials, Technical University of Darmstadt, Franziska-Braun-Str 7, 64287 Darmstadt, Germany; ukrainczyk@wib.tu-darmstadt.de; Tel.: +49-6151-16-22214

**Keywords:** geopolymers, metakaolin purity, alkali leaching, acetic acid attack, mathematical modeling, apparent diffusion coefficient

## Abstract

This paper investigates alkali leaching from geopolymers under various concentrations of acetic acid solutions. The effects of the raw metakaolin purity as well as fly ash-based geopolymer mortars and pastes are considered. A new methodology for (acetic) acid attack is proposed, adapting standard approaches, where the concentration of the leached alkali in the exposure solution is measured over time. The applicability of a simple diffusion-based mathematical model to determine the apparent diffusion coefficient (*D*_app_) for geopolymer pastes and mortars was validated. At the end of the paste tests, microstructural alterations of the specimens’ cross-sections were analyzed microscopically, revealing occurrence of degradation across the outermost surface parts and, especially under acid attack, the formation of long cracks that connect the surface with the intact inner zone. Drastically different *D*_app_ are discussed in terms of the differences in the mix designs, principally resulting in different alkali-binding capacities of the geopolymers, while the acid promoted dissolution and increased porosity. As a result of this interpretation, it was concluded that *D*_app_ is governed mainly by the chemistry of the alkali release from the gel, as it overruled the effects of porosity and cracks.

## 1. Introduction

Geopolymers are upcoming inorganic binders with great potential for niche applications in concrete structures [1,2,3], particularly where strong resistance against organic acids [4,5,6,7] as well as mineral acids [8,9,10,11,12,13,14,15] is required. A low calcium content in geopolymers represents a vital feature [4] and separates them from a broader class of alkali-activated binders. Geopolymer binders harden by the polycondensation (called geopolymerization) reaction of aluminate and silicate tetrahedrons. Those building blocks are cross-linked via oxo-bridging covalent bonds, resulting in a negative charge of aluminate units within polymeric chains, which are compensated by alkali cation precursors. The gel structure has randomly oriented aluminate and silicate polymeric chains, or, more precisely, their association exhibits no long-range order. This amorphous zeolite-like molecular structure explains the stability of the inorganic polymeric framework, which may allow for leaching of alkalis via a cation exchange mechanism [10,16,17], which is based on retaining the negative charge compensation with other cation species. However, below some critical pH [18], the acid attack may significantly dissolve the gel structure, resulting in increased leaching and degradation of material. Both leaching of alkalis and the solubility of the gel framework play a crucial role in the leaching [16,17,18,19,20], acid attack [5,7,10,15] and waste stabilization [20,21] performance of geopolymers. Moreover, they may reduce the alkalinity of the pore solution [7,11,14,17], thus inducing possible corrosion of reinforcing steel in concrete structures [1,19]. Concrete infrastructures can be exposed to organic acids in many industrial environments such as agricultural (animal feed, animal waste, anaerobic digestion and crop storage), wine, sugar, dairy and waste management. The aggressiveness of such aqueous media is affected by the solubility and expansion degree of acid salts, and for cement (i.e., high-calcium)-based binders, it increases in the following order: oxalic < tartaric < acetic < lactic [22]. When the acid salt is soluble, the aggressiveness is mainly related to the acid dissociation constant (i.e., p*K*_a_ value). It should be noted that the term weak acid seems confusing here, as it does not refer to the aggressiveness, but to the category of acid dissociation, namely, where the H^+^ cations can be weakly or strongly dissociated from the acid molecule.

An overview of a wide range of accelerated test methods that can be used to determine the resistance of concrete to organic acid attack is summarized in [23]. Guidelines provided in the ASTM C267-01 standard [24] are based on measurements of mass and compressive strength loss of mortar (50 mm) cubes or cylinders (*H* = *D* = 2.5 cm; or *D*_min_ = 5 cm and *H* = 2*D*) following immersion in acid solutions (after 1, 7, 14, 28, 56 and 84 days). The (*V_l_/S*) ratio of the acid solution volume (*V_l_*) to specimen surface area (*S*) is not specified as they should include accelerated exposure conditions that are realistic for various practical field applications. Therefore, different adaptations of the testing procedures can be found in the literature, e.g., the organic acid solution can be titrated to maintain its pH [4,23] or cyclically replenished [5,6,7], where the time intervals may also vary. The total number of specimens (and even types) per container is also variable, although a single specimen would be preferential to avoid any interactions among them (and especially the different mix types).

Powder precursors for production of geopolymers include a wide variety of aluminosilicate-rich raw materials. Their composition and reactivity have a major influence on the properties of the final product. One of the most decisive parameters is the (reactive) Si/Al ratio of the raw material composition which affects, among other things, the geopolymerization rate [25], the strength [26], the alkali leaching [20] and the acid resistance [4,9,11,14] of a geopolymer. Koening et al. [4] studied the effect of slag (i.e., calcium) dosage to fly ash-based geopolymers on degradation induced by organic acids at pH 3, as a representative exposure condition in agricultural constructions. They found that the degradation due to the organic acids increases with increasing calcium content in the geopolymer gel. Therefore, in high-calcium binders such as cement-based and high-calcium alkali-activated materials, acetic acid attack results in a high-porosity surface layer due to dissolution of the highly soluble acetate salts, complexation-enhanced solubility of Ca-rich (and Al-rich) phases and pH buffering characteristics. Aiken et al. [6] also confirmed that fly ash-based geopolymers had a greater resistance to acetic and lactic acids than Portland cement, supported by smaller mass losses. This was ascribed to the higher stability of the geopolymer gel in organic acids compared to dissolvable Ca-rich cement systems, supported by a higher porosity in geopolymers that enables easier inward diffusion of the acid. Ukrainczyk et al. [5] found that deterioration depths of mortars due to acetic acid attack increased in the order geopolymer < calcium aluminate < Portland cement, where the degradation rate on the geopolymer was at least half of the other two cement types.

From an exposure (boundary) condition point of view, the rate of leaching is influenced by the amount and mobility (running/still) of water on the surface of the material as well as the chemistry, e.g., pH of the solution. Mobility leaching scenarios may include a constant (still) contact leaching of water/solution (e.g., underground water on foundations); intermittent contact (e.g., seepage water on foundations, rain and/or condensation on facades); and flowing water (e.g., shotcrete on tunnel liners, running groundwater around concrete foundations). In concrete design practices (e.g., fib 2020, durability exposure classification CIA Z7/02 2018, standard EN 206-AS/NZS 4058), acid resistance is categorized in exposure classes for acids: mild (XA1), medium (XA2), severe (XA3) and very severe (XA4). For exposure to acidic conditions in the ground, those classes are related to ranges in pH values and soil type, where the pH range shifts to lower values by 0.5 pH units from sandy/flowing to medium to clay/stagnant soil types. The size of a concrete structural component limits the total amount of leachable species. For small concrete total volume sizes, depletion of leached species in concrete progressively lowers the leaching rate. The leaching potential of monolithic (concrete) materials can be evaluated in terms of the cumulative leaching (e.g., in mg/m^2^) according to standards CEN 15863:2015 [27] and CEN/TS 16637-2:2014 [28] as well as ASTM C1308-08 [29]. In ASTM C1308-08, the depletion effect is considered by presenting the results in terms of cumulative fraction leached (*CFL*) values, where the leached amounts are normalized to the initial total amount of a particular species in mg per specimen which can be leached. In some similar standards (e.g., [30]), the *CFL* values are furthermore multiplied by the *V*/*S* ratio (i.e., the volume of the specimen *V* normalized to the surface area of the sample *S*) and thus have a unit in cm [21]. Here, the use the ASTM definition of *CFL* is preferred as it emphasizes the depletion effect (e.g., for *CFL* < 0.2), although the change in this definition would not affect the end modeling results if the change is correctly implemented in a model (see below Equation (2)).

Recently, Sun and Vollpracht [20] employed the CEN/TS standard [28] to study the leaching of fly ash- and metakaolin-based geopolymer mortars in pure water, concluding that the leaching of species is controlled by a diffusion mechanism. However, the effect of the acid environment on the alkali leaching from the geopolymer is still not clear. Moreover, no mathematical modeling approaches were used to calibrate the apparent diffusion coefficients and test modeling assumptions. If the *CFL* values predicted by the diffusion-based model agree with the measured values within a criterion related to the uncertainty of the regression, as proposed in the ASTM C1308-08 standard [29], then it can be concluded that diffusion is the rate-determining process in the leaching mechanism. If positively evaluated, the diffusion model can be used to calculate releases over long times at the same temperature and boundary conditions. However, they would also provide first approximations for engineering extrapolations to different boundary conditions, which are of practical relevance, e.g., in construction materials exposed to various acidic conditions, although, scientifically, they would still require further validations.

Based on the aforementioned research gaps in the existing literature, this paper presents some results on alkali leaching from geopolymers, particularly focusing on the effects of raw materials for production of geopolymers (purity of metakaolins vs. fly ash pastes and mortars) and pH of the exposure solutions, namely, pure water and various concentrations of acetic acid. Moreover, the applicability of a simple diffusion mathematical model is discussed and validated to determine the apparent diffusion coefficient of geopolymer pastes and mortars. For this, standard methodologies [29,30] for leaching in pure water are adjusted to adapt them to more aggressive conditions met in acidic solutions. The concentration of eluted alkali elements in the exposure solution was measured, while microstructural alterations of the specimens’ cross-sections were analyzed microscopically. Two types of geopolymer pastes studied are based on mix designs using pure and quartz-rich metakaolins. Finally, in the Discussion Section (Section 4), the results and findings on paste samples are extended to mortar samples adapted from recent literature results [20] of standard leaching measurements in pure water, which are based on (comparable) metakaolin as well as fly ash precursors.

## 2. Materials and Methods

### 2.1. Raw Materials

Chemical composition of the raw materials used for preparation of geopolymers is given in Table 1. For the preparation of geopolymer specimens, two industrial types of metakaolins were used: a high-purity metakaolin (MK2, a commercial calcined clay originating from secondary geological deposits) and a quartz-rich one (MK1, a commercial calcined clay originating from primary geological deposits). The used metakaolins are commercial products of industrial-scale calcination and grinding. MK1 was calcined in an industrial rotary kiln for about 4 h at 700–750 °C with a production capacity of about 11 tons/h. MK2 was calcined in a multiple-hearth (Herreshoff) furnace, where in each hearth (unlike in rotary kilns), the calcination temperature and time were precisely controlled (not known but could be assumed to be <~750) to ensure high reactivity. Quantitative powder X-ray diffraction by means of DIFFRAC.TOPAS (Version 5, Bruker, Billerica, MA, USA) software for Rietveld refinement and using 10 wt.% spiked corundum resulted in 81 wt.% amorphous and 10 wt.% quartz in the MK2 metakaolin, while 50 wt.% amorphous and 40 wt.% quartz in the MK1 metakaolin. Metakaolin had a Blaine specific surface area of 26,000 and 10,000 cm²/g and a median grain size of 6 and 41 µm for K2 and K1, respectively. Potassium silicate solution was used as alkaline activator, with a molar SiO_2_/K_2_O ratio of 1.5, 45% total SiO_2_ and K_2_O “solid” content, 20 mPas viscosity and 1.51 g/cm³ density. Ultra-pure deionized water, having resistivity >18.1 M·Ohm·cm, was used as pure leaching solution. Acetic acid solutions were prepared by diluting the glacial acetic acid (100% extra pure, Carl Roth, Karlsruhe, Germany).

### 2.2. Geopolymer Specimen Preparation and Basic Properties

Geopolymer pastes were prepared with K-waterglass to a metakaolin mass ratio of 1.0 for MK2 and 0.8 for MK1 (reasons for this ratio are discussed at the beginning of the Results Section (Section 3)). The workability of fresh geopolymer paste was measured according to DIN EN 1015-3 by the spread flow test, but without operating the spindle of the table. The compressive strength of the geopolymer paste was measured according to EN 1015–11 on a specimen size of 160 mm × 40 mm × 40 mm, but without use of sand aggregates. The porosity was measured by water absorption of samples dried at 50 °C till reaching constant mass.

After mixing the geopolymer, fresh pastes were molded in plastic cylinders (*D* = 8.5 mm and *H* = 3 cm) and cured at room temperature for 28 days. Cured specimens were embedded in epoxy resin. Epoxy provided good adhesion with the geopolymer, enabling good sealing of the interface, also in the axial direction. To avoid epoxy impregnation into the geopolymer pore system, no vacuum or specimen pre-drying was used. After the resin was cured, each specimen was sectioned to 2 cm height using a low-speed diamond saw to expose one base of the cylinder to a leaching solution. In this way, one-dimensional leaching conditions were experimentally assured.

To limit carbonation effects during the specimens’ preparation, leaching experiments and sample storage, nitrogen air flow was used. For example, this is needed when opening the curing or leaching containers, especially during sampling of the replenishment solutions (described in the next section). Containers in which the solid specimens were stored were purged with nitrogen flow and sealed, while the gas environment was regulated by concentrated KOH solution to bind any remaining/diffusing CO_2_.

### 2.3. Leaching (in Acid) Experimental Setup

Diffusion and dissolution of potassium from the geopolymer paste were investigated by immersing geopolymer cylindrical specimens (*D* = 8.5 mm, *H* = 2 cm) in ultra-pure water and 100, 10 and 1 mmolar (mM) acetic acid by replenishing the leaching solution over time. Experimental one-dimensional diffusion conditions were assured by exposing the one base of the cylindrical specimen, while the other surfaces of the cylinder were sealed by epoxy resin (described in Section 2.2). An overview on the experimental plan for the leaching tests is given in Table 2. Specimen notation used indicates the type of metakaolin used in the geopolymer mix design, i.e., pure (M2) or quartz-rich (M1), and the type of exposure condition, i.e., H0 for ultra-pure water and A2, A3 and A4 for acetic acid in a concentration decreasing order, where the numbers represent a pH class (one-digit rounded numbers of measured pH presented in Results Section (Section 3)) of the initial solution. Prepared initial solutions had pH values of 2.8, 3.4, 3.9 and neutral to mimic various (acidic) exposure conditions in agricultural (pH ~ 3 [4]) and underground constructions (e.g., see durability exposure classification in fib 2020, CIA Z7/02 2018 and standard EN 206-AS/NZS 4058).

Specimens were immersed in 100 mL of 0, 1, 10 and 100 mM acetic acid maintained at 21 °C. After 1–56 days of exposure, in 10 time intervals (1, 3, 7, 14, 21, 28, 35, 42, 49, 56 days), the acetic acid solution was replaced with a new one (meaning free of leaching species). During renewals of the leaching solution, the specimen was exposed to air for as short a time as possible and put into a new leaching solution. The time intervals were chosen according to the semi-dynamic leaching method for solidified waste [29,30]. The acidic solution was replenished to maintain the boundary condition, and to sample the leached potassium. The initial high acid concentration chosen (100 mM) was adopted for this study to account for the acid depletion by the acid–base reaction with the geopolymer. The specimens were immersed in the leaching solution, contained in closed polyethylene cylindrical cups (*D* = 5 cm). The cups were rotated continuously during the whole duration of the leaching experiment, disrupted only for measurements and solution sampling and replacement at the specified time intervals. Rotation speed was maintained at 0.5 Hz by a chemical laboratory rotation device.

Two separate eluate solution samples were taken: one for ICP-MS measurements, and another for immediate pH measurement using a pH electrode (Hanna pH 211, Bayern, Germany). The eluates were filtered (several times) through a 0.45 µm cellulose acetate membrane and diluted for ICP-MS analysis. The filtered liquid samples were analyzed by inductively coupled plasma mass spectrometry (ICP-MS) conducted in an accredited laboratory (according to DIN EN ISO/IEC 17025) following the EN ISO 17294-2: 2005-02 standard method. Measurement error was <1% for concentrated eluates leached in 0.1 M acetic acid and <5% for an order of magnitude lower concentrations in eluates leached in pure water, while the potassium detection limit was 50 μg·L^−1^.

### 2.4. Mathematical Modeling

Following the semi-dynamic leaching method standard [29], a cumulative fraction leached (*CFL*) of a species from a solidified material specimen surface area in constant contact with demineralized water was specified by

(1) $$CFL_{n}=\sum_{i=1}^{n}IFL_{n}=\sum_{i=1}^{n}\frac{c_{i}}{C_{0}}$$

where:

- *CFL_n_* is the cumulative fraction of species leached for a period *n* including all individual fractions *i* = 1 to *n*;
- *IFL_n_* is the incremental fraction of species leached during test interval *n*;
- *c*_i_ is the concentration of the species in eluate for the incremental test interval *n* in mg units (related to the leaching test setup, i.e., the leaching solution volume);
- *C*_0_ is the (total) concentration of the species in the specimen at the beginning of the test, in mg units (per specimen).

The ASTM C1308-08 [29] determines if the leaching mechanism is controlled by mass diffusion, calibrating values of the diffusion coefficient based on mathematical diffusion models based on Fick’s second law. The analytical solution of Fick’s second law (partial differential equation) for diffusion from a semi-infinite solid can be easily calculated from the following expression (Equation (2)) [29]:

(2) $$CFL=2\frac{S}{V}\sqrt{\left(\frac{D_{\mathrm{app}}\;t}{\pi }\right)}$$

where:

- *D*_app_ is the apparent diffusion coefficient in cm^2^/s (in [29], it is named effective; see Discussion Section (Section 4));
- *t* is the duration of exposure in s;
- *S* is the surface area of the specimen in cm^2^;
- *V* is the specimen volume in cm^3^.

The value of the effective diffusion coefficient is calibrated by regressing the analytical model (Equation (2)) to the *CFL* measurement data obtained from the leaching test results. Mathematical regression analysis was performed using the Levenberg–Marquardt method of optimization in software Origin Pro 2015. The percent relative error in the fit of the model to the data (*rel. error*) was determined according to [29] as

(3) $$rel.\;error=\frac{\sum_{i=1}^{N}{\left(CFL_{i,\;model}\;-\;CFL_{i,\;measured}\right)}^{2}}{CFL_{N,\;measured}}\;100\%$$

where *CFL_N,measured_* is the measured *CFL* value of the experimental data for the longest duration, and in our experimental test case, *N* = 10 represents the duration of 56 days.

### 2.5. SEM-BSE Microscopy on Polished Cross-Sections

The polished cross-sections of the 56-day-aged leached specimens were imaged by environmental scanning electron microscopy using a back-scattered electron detector (SEM-BSE, Zeiss EVO LS25, Oberkochen, Germany). SEM operating conditions were 1.1 nA (probe spot current) and 13 kV under low-vacuum mode (10 Pa) to prevent charging effects on the samples (no conductive coatings were needed). The cut cross-sections were first dried (at 50 °C in a ventilated oven for 1 day) and then impregnated with low-viscosity (nominally 0.6 mPa s) liquid epoxy resin (EPOFIX from Struvers, Ballerup, Denmark) using a vacuum impregnation device (CitoVac from Struvers, Ballerup, Denmark) at a pressure of 20 kPa. The sample was polished using a semi-automatic grinding–polishing machine (LaboSystem, Struers, Ballerup Denmark), initially using a resin-bonded diamond disc (hardness range HV 150 to 2000) at a rotational speed of 300 rpm. Then, it was polished to the desired level using a lubricated cloth and polycrystalline diamond spray of, consecutively, 9, 3, and 1 μm sizes at a rotational speed of 150 rpm.

## 3. Results

### 3.1. Basic Properties of Geopolymer Pastes

The workability of the fresh geopolymer paste was 10 (no self-leveling) and 33 cm spread for M2 and M1, respectively. The high difference in workability between the two paste mixtures is due to the differences in purity of the used metakaolin powder precursors, having a metakaolin content of as high as 81 wt.% in M2, while only 50 wt.% (and 40 wt.% quartz) in M1 (estimated by QXRD). Such a difference in the purity also resulted in a huge difference in the (Blaine) specific surface area of 26,000 and 10,000 cm²/g and median grain size of 6 and 41 µm for M2 and M1, respectively. Therefore, the metakaolin to waterglass ratios were selected according to the good workability and mechanical properties of the geopolymer mortars [12,13] to be around one in the M2 case in order to enable a workable paste (and mortar) and allow the majority of the metakaolin to take part in the geopolymerization reaction. On the other hand, the M1/waterglass ratio was reduced to 0.8, having a much better workability and less (pure) metakaolin to react with the waterglass partner in the geopolymerization reaction.

The compressive strength of the geopolymer pastes after 28 days was 43 ± 2 and 47 ± 2 MPa for M2 and M1, respectively, where the lower strength could be attributed to a higher porosity in the M2 (35%) than in the M1 sample (30%), in agreement with the higher waterglass/metakaolin ratio in the mix design (1.0 vs. 0.8).

### 3.2. pH and Alkali Leaching

**Figure 1:**
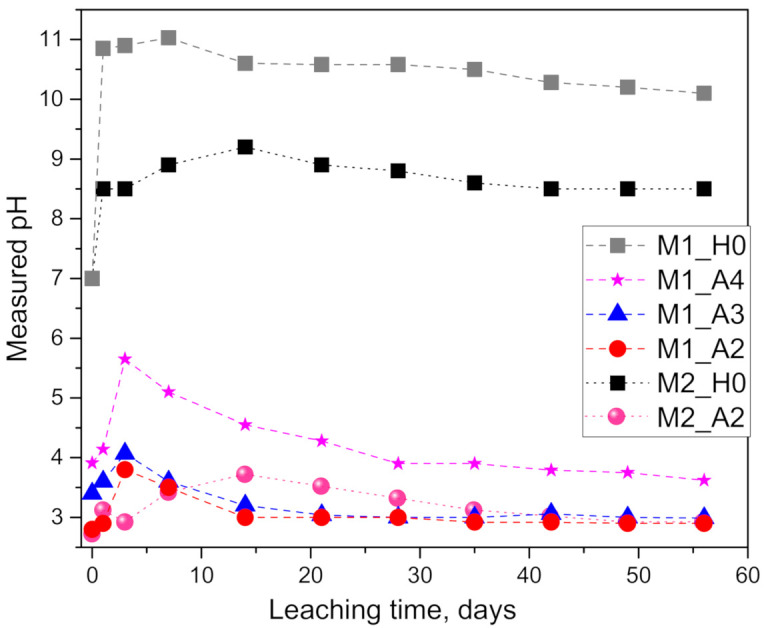
Results of pH measurements of the solutions after reaching the planned leaching intervals: the effect of geopolymer type (M1 and M2) and the concentration of the acetic acid (sample notation in Table 2).

Results of measuring the pH of the solutions (being replenished) over time are presented in Figure 1. Measured pH values of the prepared initial solutions (2.8, 3.4, 3.9) are in very good agreement with the calculated theoretical values (2.87, 3.38, 3.90) using the dissociation constant of *K_a_* = 1.8 × 10^−5^ (and solving the equation *a x* − *K_a_*(*a − x*) = 0 for *x*, where *x* and *a* are H^+^ and AcH concentrations, respectively). As a result of leaching (and dissolution) of alkaline elements from the geopolymers, the pH increased to alkaline values of above 8 and 10 in pure water or partly consumed the buffer capacity of the acetic acid, resulting in an (a much lower) increase in the pH.

In case of leaching in pure water, the significantly higher pH values reached for M1_H0 than for M2_H0 are attributed to the lower leaching rates of alkalis from the M1 geopolymer sample compared to M2 (proven later by leached K^+^ concentrations). Interestingly, the trend of those two curves in Figure 1 shows a rapid initial increase in pH followed by a shift in reaching a maximum at 7 (pH = 11.0) and 14 days (pH = 9.2) for M1 and M2, respectively, monotonically decaying subsequently. A similar shift in leaching rates can also be observed when leaching in acetic acid, where M1_A2 and M2_A2 again reach a maximum in pH after 3 and 7 days, respectively. Lower leaching rates in M2 compared to M1 can be linked to the differences in the mix designs and properties of the geopolymers (see Section 4).

Investigating the effect of the acid concentration on the M1 geopolymer, it can be observed (Figure 1) that the different pH curves (M1_A4, A3 and A2) have a similar trend but are shifted in magnitudes, where the exposure to higher acid concentrations results in lower values, as expected. In all three cases, the pH increases, reaching a maximum at 3 days, monotonically decaying subsequently.

**Figure 2:**
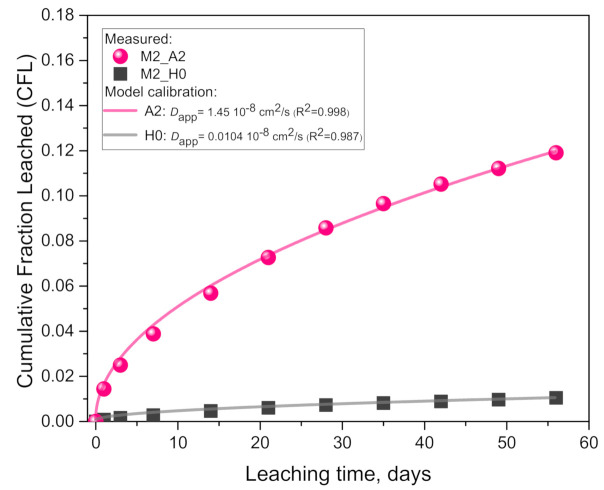
Results of cumulative fraction of leached alkali (K^+^) from geopolymer M2 in pure water (H0) and 0.1 molar acetic acid (A2): comparison of the measured data (points) with the calibrated diffusion model (lines).

Results of the cumulative fraction of leached K^+^ from geopolymer M2 (Figure 2) show a hugely higher leaching rate in 0.1 molar acetic acid (A2) compared to the pure water (H0), in agreement with the pH measurements (Figure 1). Namely, the final cumulative values after 56 days (10 solution replacements) are 32.3 mg per 100 mL acid solution and 2.83 mg per 100 mL pure water, indicating an order of magnitude (1041%) difference (or 11.4 times higher values).

A comparison of the results of the model calibration (Table 3) indicates that the *D*_app_ for the acid case is 139 times higher than in the pure water case. The very good agreement with coefficient of determination (adjusted R^2^ calculated according to Origin 2015) values very close to one (Table 3) indicates that the leaching process is limited by a (an apparent) diffusion mechanism (further detailed in the Discussion Section (Section 4)).

**Figure 3:**
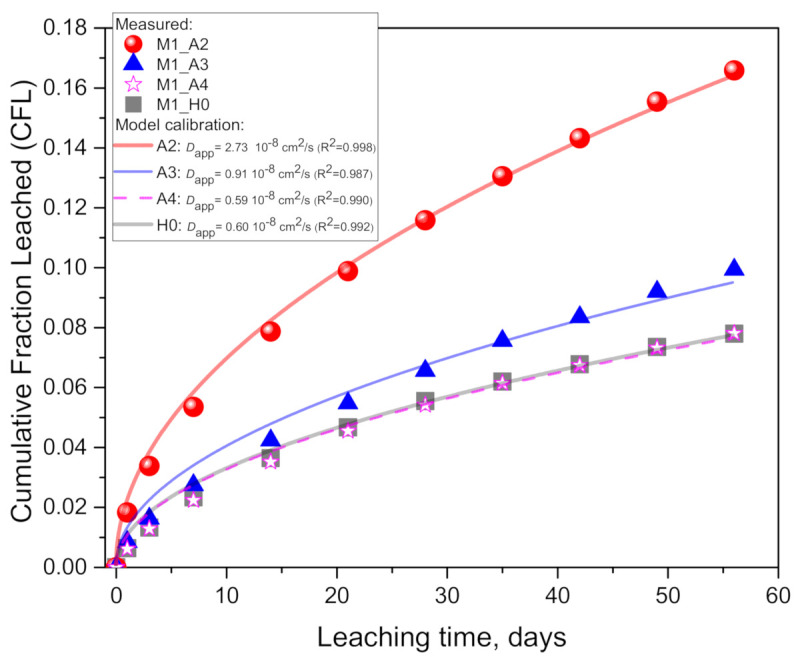
Results of the cumulative fraction of leached alkali (K^+^) from geopolymer M1: in pure water (H0) and in 1, 10 and 100 molar acetic acid (A4, A3, A2). The comparison of the measured data (points) with a mathematical model (lines) enabled evaluating the effect of the acid concentration on the calibrated apparent diffusion coefficient (Table 3).

In the M1 geopolymer case, results of the cumulative fraction of leached K^+^ in Figure 3 show the effect of the acetic acid concentration. The final cumulative values after 56 days are 40.0 (A2: 100 mM), 24.0 (A3: 10 mM), 18.8 (A4: 1mM) and 18.8 mg (H0: pure water) per 100 mL, indicating that the acid increased the potassium leaching by a 113% (for A2), 27.7% (A3) and 0% (A4) difference relative to the pure water case (H0).

A comparison of the results of the model calibration (Table 3) indicates that the *D*_app_ for the acid case is 4.5 (for A2) and 1.51 (A3) times higher than in the pure water case (H0), while having no significant difference for A4. Further, here (like in the M1 geopolymer case), a very good agreement between the model and experimental data can be observed, indicating that the leaching process is limited by a (an apparent) diffusion mechanism (see the Discussion Section (Section 4)). Coefficient of determination (R^2^) values are very close to one (Table 3) and show an increasing tendency with the acid concentration. This increase in R^2^ values agrees with a decrease in the ICP-MS measurement error for more concentrated eluates (from 5% to 1%) leached in higher concentrations of acetic acid.

Next, a comparison of the leaching rates between the M1 (Figure 2) and M2 geopolymers (Figure 3) also indicates significantly higher rates for M1, in both acid and pure water cases. In the acid case (A2), the final cumulative leaching values are 40.0 for M1 and 32.3 mg for M2, per 100 mL of leaching solution, signifying an increase in potassium leaching by 23.8%. The difference is considerably more pronounced for the pure water case (H0), where the final cumulative values are 18.8 for M1 and 2.83 mg for M2, per 100 mL of leaching solution, signifying an increase (M2→M1) in potassium leaching by as much as 564.3%. Lower leaching rates in M2 compared to M1 are explained with the differences in the mix designs (and metakaolin purity of the raw precursor) which significantly affect the properties of the geopolymers (see the Discussion Section (Section 4)). A comparison of the results of the model calibration (Table 3) also indicates the difference in the values of the calibrated *D*_app_ parameter. In the acid case (A2), the *D*_app_ value for the M1 geopolymer is 1.9 times higher than for M2, while in the pure water case (H0), the factor (M1/M2) is considerably higher, namely, 58.1.

### 3.3. SEM-BSE Microscopy

**Figure 4:**
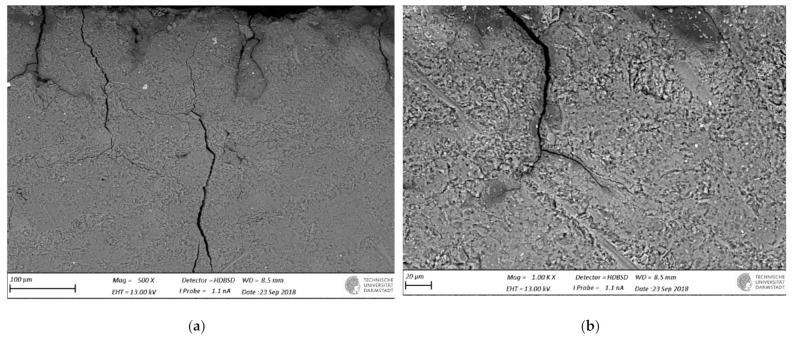
SEM-BSE images of the geopolymer **M2_H0** cross-section surface layer exposed to leaching in pure water. The water–specimen interface is in the upper side of the image: (**a**) 500× magnification and (**b**) 1000× magnification.

**Figure 5:**
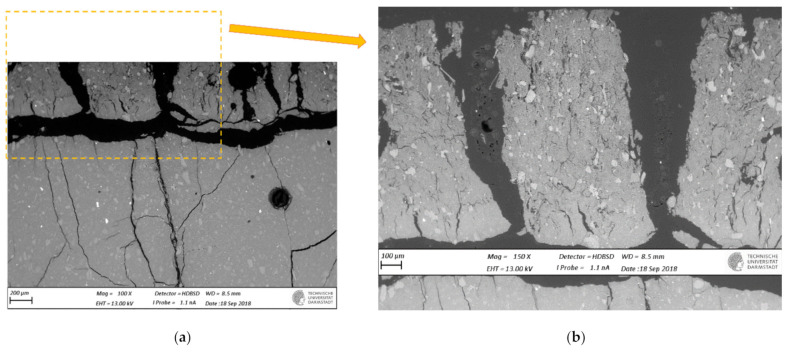
SEM-BSE images of the geopolymer **M2_A2** cross-section surface layer exposed to leaching in 0.1 molar acetic acid (the solution–specimen interface is in the upper side of the image): (**a**) 100× magnification and (**b**) 150× magnification zoom-in of image (**a**).

**Figure 6:**
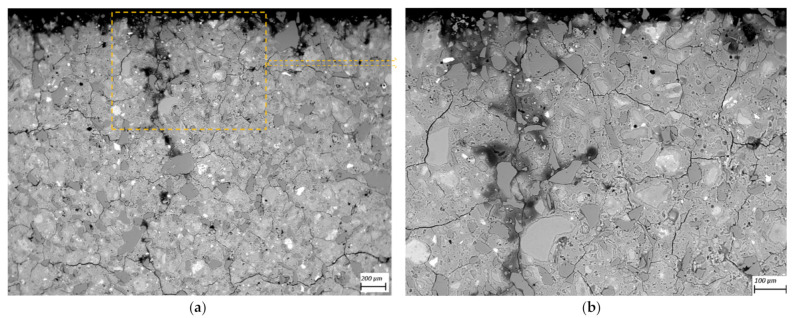
SEM-BSE images of the geopolymer **M1_H0** cross-section surface layer exposed to leaching in pure water (the solution–specimen interface is in the upper side of the image): (**a**) 100× magnification and (**b**) 250× magnification zoom-in of image (**a**).

**Figure 7:**
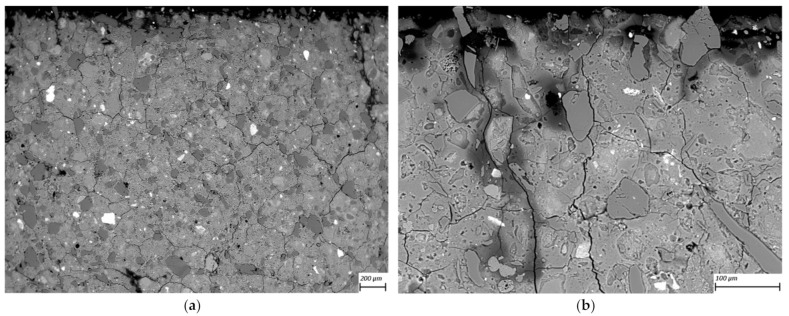
SEM-BSE images of the geopolymer **M1_A4** cross-section surface layer exposed to leaching in 1 mM acetic acid (the solution–specimen interface is in the upper side of the image): (**a**) 100× magnification and (**b**) 500× magnification zoom-in of image (**a**).

**Figure 8:**
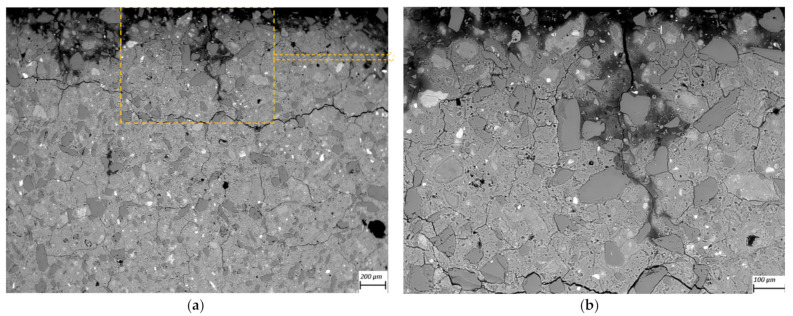
SEM-BSE images of the geopolymer **M1_A3** cross-section surface layer exposed to leaching in 10 mM acetic acid (the solution–specimen interface is in the upper side of the image): (**a**) 100× magnification and the zoom-in inset with (**b**) 250× magnification.

**Figure 9:**
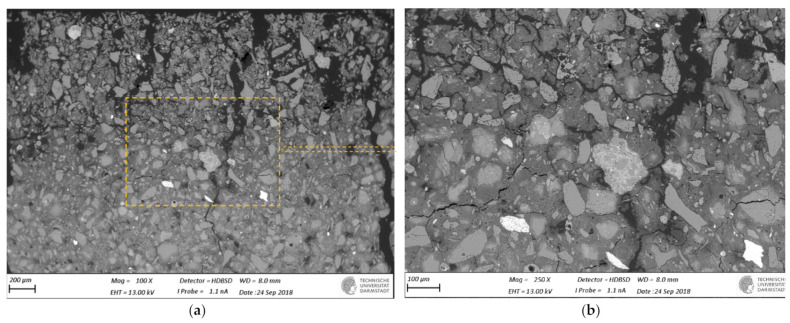
SEM-BSE images of the geopolymer **M1_A2** cross-section surface layer exposed to leaching in 10 mM acetic acid (the solution–specimen interface is in the upper side of the image): (**a**) 100× magnification and (**b**) 250× magnification zoom-in of image (**a**).

SEM-BSE images of cross-sections of leached geopolymer hardened pastes (M1 and M2) after 56 days of exposure to various concentrations of acetic acid (Figure 4, Figure 5, Figure 6, Figure 7, Figure 8 and Figure 9) revealed the deterioration degree. 

In SEM-BSE images, the grayscale brightness level is proportional to the phase proton number and thus enables separation and identification of the local microstructural phase composition [31]. The non-reacted grains from the metakaolin powder precursor have the brightest grayscale levels related to the relatively heavier atoms containing Fe– and Ti– (and Ca–) oxide phases and exhibit sharp boundaries. Quartz and SiO_2_-rich particles are less bright due to their relatively lower atomic mass, where quartz particles have clear sharp boundaries, while other silica-rich grains have non-sharp boundaries as regions with diffuse outlines, discerning the level of geopolymerization reactivity in alkaline media. The geopolymer matrix has intermediate grayscale levels composed of the K–A–S–H gel phase, which is darker for higher K and water contents. Thus, more porous geopolymer gels exhibit darker grayscale levels, while pores that are impregnated with epoxy resin have the darkest grayscale level. Thus, changes in the phase composition dominated by K^+^ leaching and porosity at the surface of the geopolymer specimens could be estimated based on (semi-)qualitative comparison of the areas in BSE images.

The outermost surface exposed to the external solution is represented by the upper edge of the SEM-EDS image. Thus, the acid always penetrated from the upper side of the images, while the diffusion of leached elements went in an upward direction. Comparison of Figure 4 (M2_H0) with Figure 5 (M2_A2) as well as Figure 6 (M1_H0) and Figure 7 (M1_A4) with Figure 9 (M1_A2) clearly shows that the deteriorated layer under acid attack was significantly more affected than under pure water (and low A4 acid concentration) exposure, as expected. The uppermost parts, near the specimen’s surface exposed to the external solution, and especially under acid attack, appeared to be highly porous (i.e., darker). The geopolymer exposed to leaching under water (and low A4 acid concentration), meanwhile, was obviously less degraded. Based on image analysis of SEM-BSE micrographs, an averaged thickness of the degradation zone (including the dissolution based on remaining quartz impurities) was estimated to be 1 mm for M2_A2 and 20 μm for the M2_H0 case. In M1 geopolymer cases, the average degradation zone was estimated to be 100 μm, 100 μm, 200 μm and 1.2 mm for H0, A4, A3 and A2 exposure cases, respectively.

The SEM images in Figure 5 (M2_A2) and Figure 9 (M1_A2) evidently reveal the occurrence of lengthy cracks that connect the top surface layer of the sound inner zone. These cracks could be attributed to the shrinkage resulting from the dissolution of geopolymer elements in harsh conditions of 0.1 molar acetic acid, as well as being due to drying shrinkage conditions used for sample preparations (detailed in Section 2.5). Comparison of Figure 4 (M1_H0), Figure 6 (M2_H0) and Figure 7 (M1_A4) with Figure 5 (M2_A2) and Figure 9 (M1_A2) shows that exposures to water (H0) and low acid concentration (A4) had by far the lowest tendency to crack formation than the samples leached in more acidic (A3 and A2) conditions. Exposure to the intermediate acid condition (M1_A3, Figure 8) resulted in the intermediate level of degradation, in agreement with the pH and K leaching results. Figure 5 (M2_A2) and Figure 9 (M1_A2) compared to all other SEM-BSE images show that the A2 case exhibited the highest degradation with a substantial cracked degradation zone, while M2_A2 showed a detachment of the top surface layer of the damaged specimen. The dissolution-induced shrinkage caused by the acetic acid attack resulted in differential stresses, putting the geopolymer paste specimen under tension. Shorter and thinner cracks formed when the tensile stress was enough to induce fracture. All SEM-BSE images (Figure 4, Figure 5, Figure 6, Figure 7, Figure 8 and Figure 9) demonstrate that the lengthy cracks have a crucial role in forming preferential diffusion leaching paths. This is more clearly observed in the zoom-in images (Figure 4b, Figure 5b, Figure 6b, Figure 7b, Figure 8b and Figure 9b, i.e., positioned on the right side), where the crack–matrix interface boundaries indicate increased regions with diffuse outlines for higher acid concentrations, discerning the level of leaching/dissolution of the surrounding geopolymer matrix. Lengthy orthogonal cracks can be observed which act as preferential leaching paths which diffuse the dissolved species towards upper surface layers and result in increased dissolution of the geopolymer matrix also in the horizontal direction, being more porous with increased concentration of the acid exposure solution. The zoom-ins on the surface layers (Figure 5b, Figure 6b, Figure 7b, Figure 8b and Figure 9b) show the random distribution of unreacted quartz (darker gray) grains as well as clay (brighter) particles embedded in the geopolymer framework, which originate from much higher amounts of impurities present as grains in the raw metakaolin MK1 (Figure 6, Figure 7, Figure 8 and Figure 9) than MK2 (Figure 4 and Figure 5), in agreement with the quantitative results of X-ray diffraction and particle sizes shown in the Materials Section (Section 2). The quartz grains show no signs of dissolution/alteration in the various degradation layers.

## 4. Discussion

### 4.1. Experimentally Supported Modeling Approach

The semi-infinitive modeling assumption is assured by the chosen specimen’s geometry (and use of epoxy coating) and the *V_l_/S* ratio of the proposed experimental leaching setup, which deviates from the standard ones [24,27,28,29,30]. This is because the modeling assumption is valid for diffusive leaching of species from porous materials that give a low cumulative leached fraction, i.e., leached-to-availability ratio of, namely, less than 0.2 [29]. This means that if more than 20% of the leached element is depleted from the (cylindrical) specimens (i.e., *CFL* > 0.2), the applicability of the model (Equation (2)) is highly questionable. Alternatively, to consider the depletion of the leaching species in the specimen due to its outwards leaching flux, the diffusion model through a finite cylinder geometry should be used. This requires implementation of much more complicated solution strategies than in Equation (2), e.g., an analytical solution for a finite cylinder geometry as given in [29]. However, as it is based on the very slow numerical convergence of infinitive open series (with Bessel function parameters), closed form analytical expressions can be used [32] to control the maximum absolute error by truncating the open series. Other specimen geometries, such as standard prismatic ones [24,27,28] or cubes [24], do not facilitate simplifying the modeling of the test results to linear diffusion and thus may require the use of 3D numerical solutions even when assuming that the *D*_app_ is constant with time (and space) and independent of the concentration [16].

The specimen size and leaching solution volume must be selected by finding the middle ground between the benefits of using a representatively big specimen and the associated difficulties in the handling of big leaching solution volumes. The specimen size is decided based on its homogeneity and ease of sampling solid materials after leaching tests. However, this size is limited by the handling convenience, analytical limitations and waste disposal of leaching solutions and solid specimens. When extending the water leaching test to model the acid attack, in particular, the limits in the leaching solution (over-)saturation in terms of solids precipitation must be considered. They could have undesired feedback effects on the diffusion mechanism and thus should be avoided. Undesired precipitations can be identified and avoided or diminished by performing tests at (differently) higher *V_l_/S* ratios, where precipitations in the leaching solution are expected to be less significant. However, this has to be compromised with wet chemistry analytics, as a too high *V_l_/S* would result in too low leaching rates to achieve measurable (accurate/precise) solution concentrations.

The *V_l_/S* ratio used on paste samples resulted in a value of 176 cm^3^/cm^2^ to avoid undesired precipitations in leaching solutions while keeping the measurements accurate (details given in the Methodology Section (Section 2)). This *S*/*V_l_* is roughly 20 times higher than the values proposed in standards, namely, 8 cm^3^/cm^2^ in [28] and 10 cm^3^/cm^2^ in [29,30], which are, anyway, not strictly defined (in [29]) but are also adjustable to achieve the compromise for a specific material’s cases.

### 4.2. Geopolymer Pastes M1 vs. M2 and Effect of Acid Concentration

The lower leaching rates in M2 compared to M1 can be linked to the differences in the mix designs and properties of the geopolymers. More specifically, the lower total amount of reactive precursors (metakaolin and waterglass) and the lower reactive Al/Si ratio due to the lower ratio of waterglass to reactive metakaolin in the M1 mix resulted in there being less geopolymer which can bind alkalis in its solid framework network. This lower alkali-binding capacity enabled higher concentrations of the free alkali in the pore solution (being in equilibrium with the geopolymer gel) which can more easily leach out due to higher (pure diffusive) concentration gradients as well as possibly being not limited by the kinetics of release of the bonded alkali. The importance of the alkali-binding capacity of the geopolymer, affected primarily by the gel amount and its Al/Si ratio, can be further emphasized by overruling the effect of increased porosity (35% for M2 compared to 30% for M1) on the leaching rates. The physical (i.e., effective diffusion, *D*_eff_) and chemical natures of the *D*_app_ can be mathematically expressed by the relationship between the two diffusion coefficients [33]:

(4) $$D_{\mathrm{app}}=D_{\mathrm{eff}}/\left(1+\frac{dC}{dc}\right)$$

where d*C*/d*c* is the alkali-binding capacity (namely, a constant, d*C*/d*c = k*, based on a linear binding isotherm, *C = k·c*) and *C* is the total (or bound) amount of alkali. Strictly speaking, the application of the (effective) diffusion model requires that the porous material remains intact and the leaching mechanism does not change with time. Based on the results of microscopy images, it is clear that this assumption is less and less valid with increasing acetic acid concentration in the exposure solution. This also suggests that the *D*_app_ should be space (degradation depth)-dependent, which is not considered in the used simple model, as it may require a numerical solution strategy. However, it should also be noted that the calibrated apparent diffusion coefficient (Equation (2), Table 3) considers a constant binding capacity (Equation (4)) for linear binding isotherms. Thus, it may suggest that a linearized binding simplification may also be valid, as nonlinearities in binding would result in time (and space) dependency of the *D*_app_. This also explains why the observed cracks and increased porosity due to matrix dissolution had no effect on the time dependency of the *D*_app_. This discussion leads (again) to the conclusion that the *D*_app_ is being governed by chemistry (the cation exchange release of bonded alkalis) and not by *D*_eff_ (porosity and pore morphology). This is in agreement with the finding in [34], where, with purely computational simulations on analogue Ca leaching from cement pastes, it was demonstrated that despite the drastically different material physical properties such as pore connectivity and effective diffusivity, the leaching kinetics was not affected as long as the amount of soluble phases was kept the same. The leaching kinetics was also not affected by the presence of cracks [34]. In case of geopolymers, a further study is needed to prove the linear binding assumption independently from the here proposed calibration values. As the *D*_app_ in model Equation (2) lumps the entire reactive transport process together, the simplified modeling (linear diffusion) approach proposed here could be interpreted as semi-empirical. The goodness of fit is evaluated with *rel. errors* obtained via Equation (3), and as the results (Table 3) are much less than the critical value of 0.5% [29], it can be concluded that the diffusion model accurately represents the data.

In future work, more advanced (numerical) modeling approaches are to be employed on the leaching results, in order to better separate the physical nature of the effective diffusion coefficient from the chemical binding isotherms, such as in [17]. Research so far has covered only empirical geopolymer tests on acid resistance [1,2,3,4,5,6,7,8,9,10,11,12,13,14,15], thus neglecting the fundamental chemical aspects behind it. Analogue mechanisms involved in dissolution of zeolites [35] demonstrate that decomposition of a network aluminosilicate in acid gradually shifts from initially congruent Si and Al dissolution to progressively preferential dissolution of Al, resulting in amorphous silica-rich gels, with gradually increasing Si/Al ratios. In this limited context, the solubility of a geopolymer gel depends on the pH, and for increasing acidic conditions, it is described here as follows:

- Ion exchange reaction between the cation X^+^ and the charge-compensating cations (Na^+^ or K^+^) of the geopolymer framework:

[Si–O–Al–O–Si…]^−^K(H_2_O)_n_^+^ + X^+^ + 4OH^−^ ↔ [Si–O–Al…]^−^X^+^ + Si(OH)_4(aq)_ + K(H_2_O)_n_^+^ (5)

- Partial dealumination dissolution of the aluminosilicate framework;
- Precipitation of acid anion salts (which, here in the acetic case, are highly soluble);
- Crystallization of (Na-based) zeolites causing a decrease in material strength;
- Dissolution and re-crystallization of the Si-rich aluminosilicate framework.

The cation species X^+^ which is exchanging with alkali cations could most likely be [16] Al(OH)_2_^+^ or Al_y_(OH)_z_^(3y+z)+^, but it could also other minor metal cations present as impurities in metakaolin raw materials, such as Fe, Ca or Na, and theoretically also the penetrating acid protons (H^+^). Moreover, another mechanism found in the literature [1,2] is associated with zeolite crystallization, which causes a decrease in material strength. This result is more commonly found in Na-based geopolymers [1,2,16].

In the experimental results, the focus was on geopolymer pastes, in order to see the effect of two geopolymer mix designs based on pure and impure raw metakaolin on the leaching behavior in water and in acetic acid having different concentrations. In future research efforts, the validity of the findings of this research on binder pastes should be upscaled to mortar and concrete specimens. In practice, the geopolymer paste is used as a binder in mortars and concretes, and thus the effects of aggregates on leaching must be considered as well. For this upscaling, standard leaching tests in water could be adapted and also extended for acid solutions.

### 4.3. Mortars vs. Pastes and Fly Ash- vs. Metakaolin-Based Geopolymers

Based on adaptation of recent literature data [20], it was possible to test the proposed diffusive modeling approach also on the EN 16637-2:2014 leaching standard (in pure water) [28] and compare the difference between fly ash- and metakaolin-based geopolymer mortars, and those test cases are re-named here as FAH0_mortar and M1H0_mortar, respectively. Moreover, they enabled comparing the paste results with the mortar ones and thus discuss the effect of aggregates. The metakaolin used in [20] belongs to a quartz-rich class (M1) with a reasonably similar chemical composition to the M1 used in this paper. The most significant differences are in the higher amount of Al_2_O_3_ (32.58% compared to 27.0% from Table 1), which may indicate a purer metakaolin content, and QXRD results also indicate an intermediate purity, i.e., between M1 and M2. Moreover, a Na- instead of a K-based silicate solution was used in [20]. However, the SiO_2_/Me_2_O (Me = K, Na) molar ratios of the silicate solutions are in both cases 1.5 with very similar solid contents (40.1% compared to 45.0%). Moreover, the mix design for metakaolin-based geopolymers is very similar, namely, using the metakaolin-to-waterglass mass ratio of 0.75 compared to 0.80 for the M1 test series.

**Figure 10:**
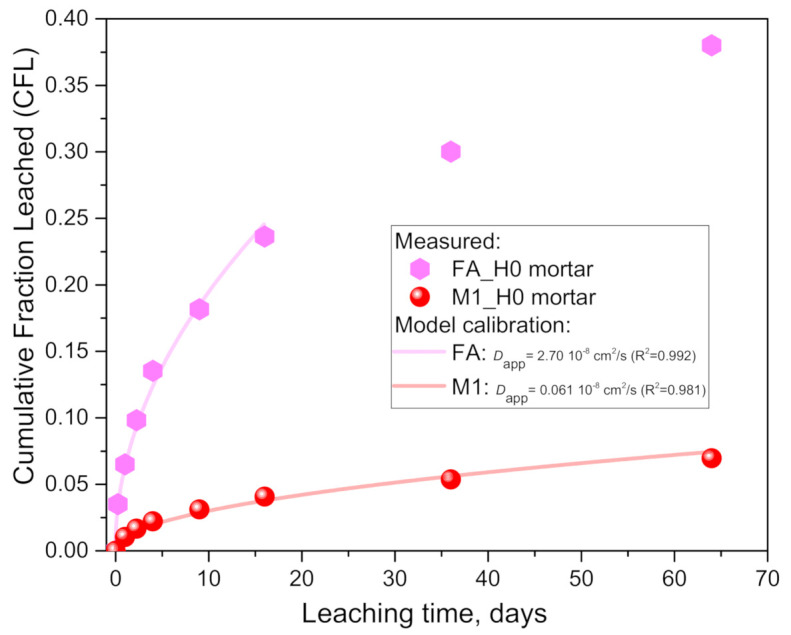
Results of cumulative fraction of leached alkali from fly ash (FA)- and metakaolin (M1)-based geopolymer prismatic mortars in pure water (H0) according to the (CEN) standard [28]. The measured cumulative fraction leached (*CFL*) values were re-calculated from data available in [20] (see text on calculation details).

First, the literature leaching results had to be transformed from the published cumulative values expressed in mg/m^2^ into the fractional ones, i.e., *CFL* values which are to be used next in modeling Equation (2). This was conducted in the following sub-steps: (1) the leached values in mg/m^2^ were transformed to mg per specimen by the surface area multiplication factor *S* (which for a 4 × 4 × 16 cm^3^ prism is 0.0288 m^2^); (2) the alkali amount in the prismatic specimen (i.e., *C*_0_ in Equation (1 and 2) and given later in Table 4) was calculated based on the given chemical composition of the anhydrous geopolymer binder and the known mix design (sand-to-binder ratio of 3 and assuming a 2 vol.% of air content). As the *CFL* values for the fly ash geopolymer mortar (FAH0_mortar) go beyond the 0.2 limit defined in ASTM C1308-08 [29] for the validity of the infinite medium modeling assumption, the model was calibrated using a limited dataset of only 0–16 days (*CFL*_(*t* = 16 days)_ = 0.236), Figure 10. The lower experimental values than the extrapolated model predictions agree with the depletion of alkalis in the finite specimen.

For the mortar cases (Figure 10 and Table 4), a very good agreement between the model and experimental data can be observed, indicating that the leaching process is limited by a (an apparent) diffusion mechanism, similar to the paste cases (Figure 2 and Figure 3, Table 3). The coefficient of determination (R^2^) values are very close to one (Table 4), and the goodness of fit quantified as *rel. error* (from Equation (3)) shows significantly lower vales than the 0.5% threshold proposed in [29], confirming that the diffusion model can accurately represent the experimental data on leaching rates.

The comparison of the adapted measured data (points in Figure 10) with a mathematical model (lines) enabled evaluating, firstly, the effect of the geopolymer type (fly ash- vs. metakaolin-based) and, secondly, the effect of the sand aggregate (mortar vs. paste) on the calibrated apparent diffusion coefficient (Table 4 vs. Table 3). The first comparison (in Table 4) indicated that the *D*_app_ for FA_mortar is 44.6 times higher than for M1_mortar. This difference is in agreement with the over four times higher leached values already reported and explained in [20] by the lower geopolymerization reaction degree of fly ash, leaving more free alkali in the pore solution (i.e., not bonded in the geopolymer gel), experimentally confirmed by extracted pore solutions [20] and in agreement with (27%) the lower compressive strength of FA_mortar than M1_mortar.

The second comparison indicated very similar *CFL* values between the mortar (M1_H0 mortar in Figure 10) and the paste specimens (M1_H0 in Figure 6); however, the 508 times higher surface areas (*S*, Table 3 vs. Table 4) in the mortar test setup resulted in much lower leaching rates (when normalized per surface area). This demonstrates that the *CFL* values of the different experimental setups are not comparable regarding the cumulative leaching amounts, as they are expressed in fractional terms and depend on the *V_l_/S* ratios. This dependence is also clearly visible in the model Equation (2). A comparison of the calibrated *D*_app_ (Table 3 and Table 4) indicated 10 times higher values for the paste (M1_H0) than for M1_mortar. The higher diffusivity in the paste is in agreement with the lower porosity and increased tortuosity (and lower connectivity and constrictivity of the pore structure [36]) due to the aggregate dilution and inclusion effects. Moreover, the geopolymer chemistry (Al/Si) and geopolymerization reaction degree could also play a significant role here. As in the fly ash geopolymers, the less reactive metakaolin may have resulted in there being more free alkalis in the pore solution which are more mobile to leach out. The lower geopolymerization reaction degrees for M1 used in the paste than in the mortar are supported by the lower Al_2_O_3_ content (more impurities in metakaolin) and 31% lower compressive strengths. The importance of the geopolymerization degree and Al/Si ratio was already well demonstrated when discussing the differences in results between M1 and M2 paste geopolymers.

## 5. Conclusions

Based on the results and discussions in this study, the following conclusions and interpretations are summarized:

1. Geopolymer paste M2 (pure metakaolin-based) exhibited an order of magnitude (1041%) higher alkali leaching in 0.1 molar acetic acid than in pure water, while the calibrated *D*_app_ was 139 times higher.
2. In the M1 (quartz-rich metakaolin-based) geopolymer paste, the potassium leaching in acetic acid increased by 113% (100 mM acid), 27.7% (10 mM) and 0% (1 mM) relative to the pure water case. The corresponding *D*_app_ for the acid case was 4.5 (100 mM) and 1.51 (10 mM) times higher than in the pure water case while having no significant difference for the 1 mM case.
3. Geopolymers based on less pure metakaolin M1 resulted in 23.8% (in 100 mM acid) and as much as 564.3% (in pure water) higher alkali leaching compared to the (pure) M2 case. The *D*_app_ value for the M1 geopolymer is 1.9 (in 100 mM acid) and 58.1 (in pure water) times higher than for M2.
4. SEM-BSD microscopy revealed occurrence of degradation and dissolution zones across the outermost surface depths, the estimated average thicknesses being for the M2 case 1 mm (in 100 mM acid) and 20 μm (water), while 100 μm (water and 1mM), 200 μm (10 mM) and 1.2 mm (100 mM) in the M1 case. Especially under acid attack, geopolymer surface layers exhibited long cracks that connect the top surface layer with the sound inner zone.
5. Lower leaching rates in (pure) M2 compared to (quartz-rich) M1 were discussed in terms of the differences in the mix designs and (mechanical, porosity and chemical) properties of the geopolymers. Here, the major importance of the alkali-binding capacity on *D*_app_ overruled the effect of increased porosity for M2 compared to M1 on the leaching rates. This also explains why the observed cracks and increased porosity due to matrix dissolution had no effect on the time dependency of *D*_app_, which led to the conclusion that the *D*_app_ is being governed by the chemistry (of the cation exchange release of bonded alkalis, Equation (5)) and not by *D*_eff_ (porosity and pore morphology).
6. Adaptation of standards ([28,29] for leaching in pure water) to evaluate the (acetic) acid attack of geopolymers was proposed and discussed in detail. The proposed diffusive modeling approach was adapted and validated on geopolymer pastes and mortars.
7. The diffusion model can accurately represent the experimental data for both pastes and mortars, evaluated by *rel. error* (Equation (3) for a goodness of calibration), being much less than the critical value of 0.5% (according to [29]).
8. *D*_app_ for fly ash mortar was 44.6 times higher than for the M1 mortar, in agreement with conclusion point 5 that a lower geopolymerization reaction degree (of fly ash [20]) results in more free alkalis in the pore solution.
9. The ten times higher *D*_app_ for the leaching of the M1 paste than the mortar (in water) could be explained by a) the lower porosity due to the aggregate inclusion (pore tortuosity, etc.) and matrix dilution effects, and b) the higher alkali-binding capacity expected from the higher geopolymerization reaction degree (and Al/Si) due to the use of purer M1 metakaolin in the paste case (in agreement with conclusion points 5 and 8).

In future work, more advanced (numerical) modeling approaches would separate the physical nature of the effective diffusion coefficient from the chemical binding isotherms. However, this also requires more research on the effective diffusive properties and thermodynamics of alkali leaching binding isotherms, as well as on dissolution of geopolymer gels as a function of pH.

## Figures and Tables

**Table 1:** Chemical composition (mass %) of metakaolins and K-based silicate solution (waterglass).

Material	SiO_2_	Al_2_O_3_	CaO	TiO_2_	Fe_2_O_3_	MgO	Na_2_O	K_2_O	H_2_O
Metakaolin MK2	50.4	40.5	0.1	1.5	2.0	0.1	0.1	0.1	–
Metakaolin MK1	67.0	27.0	1.0	1	4	0.1	0.1	0.2	–
K-waterglass	22	–	–	–	–	–	–	23	55

**Table 2:** Experimental plan for one-dimensional diffusion leaching test on geopolymer paste specimens.

Specimen Notation	AcH Concentration, mM	Solution Replenishment (pH and K^+^ Measured), Days	SEM-BSE Imaging after, Days
M1_H0	0 (ultra-pure water)	1, 3, 7, 14, 21, 28, 35, 42, 49, 56	56
M1_A2	100		
M1_A3	10		
M1_A4	1		
M2_H0	0 (ultra-pure water)		
M2_A2	100		

**Table 3:** A list of model parameters (Equation (2)) and calibration (statistical) results of alkali leaching from geopolymer paste specimens.

Sample Name	*D*_app_ 10^8^, cm^2^/s	*Std. Error* 10^8^, cm^2^/s	*R-Square*	*Rel. Error*%	*S*, cm^2^	*V*, cm^3^	*C*_0_, mg
M2_H0	0.0104	0.0004	0.987	0.017	0.567	1.418	270.8
M2_A2	1.45	0.023	0.998	0.033			
M1_H0	0.604	0.018	0.992	0.081			241.0
M1_A4	0.592	0.020	0.990	0.104			
M1_A3	0.912	0.036	0.987	0.159			
M1_A2	2.73	0.042	0.998	0.044			

**Table 4:** A list of model parameters (Equation (2)) and calibration (statistical) results for alkali leaching from geopolymer mortar prismatic (4 × 4 × 16 cm^3^) specimens in pure water.

Sample Name	*D*_app_ 10^8^ (cm^2^/s)	*Std. Error* 10^8^ (cm^2^/s)	*R-Square*	*Rel. Error* (%)	*S* (cm^2^)	*V* (cm^3^)	*c*_0_ (mg)
M1H0_mortar	0.0606	0.0036	0.981	0.101	288	256	9780
FAH0_mortar	2.70	0.113	0.992	0.085	288	256	7040

## Data Availability

Data sharing is not applicable for this article.
